# PCB pollution continues to impact populations of orcas and other dolphins in European waters

**DOI:** 10.1038/srep18573

**Published:** 2016-01-14

**Authors:** Paul D. Jepson, Rob Deaville, Jonathan L. Barber, Àlex Aguilar, Asunción Borrell, Sinéad Murphy, Jon Barry, Andrew Brownlow, James Barnett, Simon Berrow, Andrew A. Cunningham, Nicholas J. Davison, Mariel ten Doeschate, Ruth Esteban, Marisa Ferreira, Andrew D. Foote, Tilen Genov, Joan Giménez, Jan Loveridge, Ángela Llavona, Vidal Martin, David L. Maxwell, Alexandra Papachlimitzou, Rod Penrose, Matthew W. Perkins, Brian Smith, Renaud de Stephanis, Nick Tregenza, Philippe Verborgh, Antonio Fernandez, Robin J. Law

**Affiliations:** 1 Institute of Zoology, Zoological Society of London, Regent’s Park, London NW1 4RY, UK; 2 Centre for Environment, Fisheries and Aquaculture Science, Pakefield Road, Lowestoft, NR33 0HT, UK; 3 Department of Animal Biology and Biodiversity Research Institute (IRBio), University of Barcelona, Barcelona, Spain; 4 Scottish Marine Animal Stranding Scheme, SRUC Veterinary Services Drummondhill, Stratherrick Road, Inverness, IV2 4JZ, UK; 5 Environment and Sustainability Institute, University of Exeter, Cornwall Campus, Penryn, Cornwall TR10 9EZ, UK; 6 Marine and Freshwater Research Centre, Galway-Mayo Institute of Technology, Galway, Ireland; 7 CIRCE, Conservation, Information and Research on Cetaceans, Cabeza de Manzaneda 3, Algeciras, 11390, Spain; 8 Marine Animal Tissue Bank – Portugal, Soc. Portuguesa de Vida Selvagem, Dep. Biologia - Univ. do Minho & CESAM, Portugal; 9 Evolutionary Biology Centre, Uppsala University, Norbyvägen 18D, SE‐752 36 Sweden; 10 Morigenos-Slovenian Marine Mammal Society, Kidričevo nabrežje 4, 6330 Piran, Slovenia; 11 Institute for Biodiversity Studies, Science and Research Centre, University of Primorska, Koper, Slovenia; 12 Department of Biodiversity, Faculty of Mathematics, Natural Sciences and Information Technologies, University of Primorska, Koper, Slovenia; 13 Department of Conservation Biology, Estación Biológica de Doñana (EBD-CSIC), Americo Vespuccio S/N, Isla Cartuja, 42092, Sevilla, Spain; 14 Cornwall Wildlife Trust Marine Strandings Network, Five Acres, Allet, Truro, TR4 9DJ, UK; 15 Coordinadora para o Estudio dos Mamíferos Mariños (CEMMA), Apartado 15, 36380 Gondomar, Pontevedra, Spain; 16 Sociedad de Estudios de Cetáceos en Canarias (SECAC), Lanzarote, Spain; 17 Marine Environmental Monitoring, Penwalk, Llechryd, Cardigan, Ceredigion, SA43 2PS, UK; 18 The Natural History Museum, Cromwell Road, London SW7 5BD, UK; 19 Facultad de Veterinaria, Universidad de Las Palmas de Gran Canaria, Gran Canaria, Spain

## Abstract

Organochlorine (OC) pesticides and the more persistent polychlorinated biphenyls (PCBs) have well-established dose-dependent toxicities to birds, fish and mammals in experimental studies, but the actual impact of OC pollutants on European marine top predators remains unknown. Here we show that several cetacean species have very high mean blubber PCB concentrations likely to cause population declines and suppress population recovery. In a large pan-European meta-analysis of stranded (n = 929) or biopsied (n = 152) cetaceans, three out of four species:- striped dolphins (SDs), bottlenose dolphins (BNDs) and killer whales (KWs) had mean PCB levels that markedly exceeded all known marine mammal PCB toxicity thresholds. Some locations (e.g. western Mediterranean Sea, south-west Iberian Peninsula) are global PCB “hotspots” for marine mammals. Blubber PCB concentrations initially declined following a mid-1980s EU ban, but have since stabilised in UK harbour porpoises and SDs in the western Mediterranean Sea. Some small or declining populations of BNDs and KWs in the NE Atlantic were associated with low recruitment, consistent with PCB-induced reproductive toxicity. Despite regulations and mitigation measures to reduce PCB pollution, their biomagnification in marine food webs continues to cause severe impacts among cetacean top predators in European seas.

The severe impact on top predators of the bioaccumulation of DDT and other organochlorine (OC) pesticides in food webs was first identified in the early 1960s^1^. Most OC pollutants were banned in developed countries in 1970s–1980s and some terrestrial wildlife populations recovered^1^,^2^,^3^. Experimental studies have established that OC pesticides and the more persistent polychlorinated biphenyls (PCBs) have a range of species-specific and dose-dependent toxic effects such as immunosuppression and reproductive impairment in all mammalian species tested, including marine mammals^4^,^5^,^6^,^7^. However, the impact of historic and current exposure to marine pollutants on European cetacean top predators is still largely unknown.

To investigate this issue we conducted a meta-analysis of European data on summed 18–25 chlorobiphenyl congeners (∑PCB) concentrations (mg/kg lipid weight (lw)) in cetaceans that were stranded and necropsied or free-living and biopsied. Data came from 1,081 animals of four species:- harbour porpoise (*Phocoena phocoena*, HP)(n = 706), striped dolphin (*Stenella coeruleoalba*, SD)(n = 220), bottlenose dolphin (*Tursiops truncatus*, BND) (n = 131) and killer whale (*Orcinus orca*, KW)(n = 24). The ∑PCB was mainly derived from two different laboratories, but with both using internationally standardised methodology enabling full integration of data. The analysis included statistical evaluation of long-term temporal trends in ∑PCB concentrations in UK-stranded HPs (1990–2012) and Mediterranean SDs (1990–2009). We compared mean ∑PCB concentrations in all four species with a range of established PCB toxicity thresholds for marine mammals^5^,^6^,^7^. The lowest PCB toxicity threshold was a widely used and relatively low PCB toxicity threshold for onset of physiological impacts and equivalent to 9 mg/kg lipid as ∑PCB^6^ in this study. We also used the very highest PCB toxicity threshold reported for marine mammals, for profound reproductive impairment in ringed seals (*Phoca hispida*) in the Baltic Sea, and equivalent to 41mg/kg lipid as ∑PCB in this study^6^.

We compared mean PCBs concentrations in UK-stranded HPs that were “healthy” and died of acute physical trauma (n = 345) (control group) with mean PCBs concentrations in HPs that died of infectious diseases (n = 252) (case group) or starvation (n = 42). Where data permitted, sex differences in mean PCB concentrations were investigated to assess maternal offloading of PCBs via lactation. Finally, we reviewed the current population and reproductive status of the extant odontocete populations in European waters using systematic local and international observational survey and other data collected over the past 15–20 years (e.g. *International Union for Conservation of Nature* assessments)^8^.

## Results

### Spatial and temporal trends and sex differences in ∑PCB exposure

**Figure 1:**
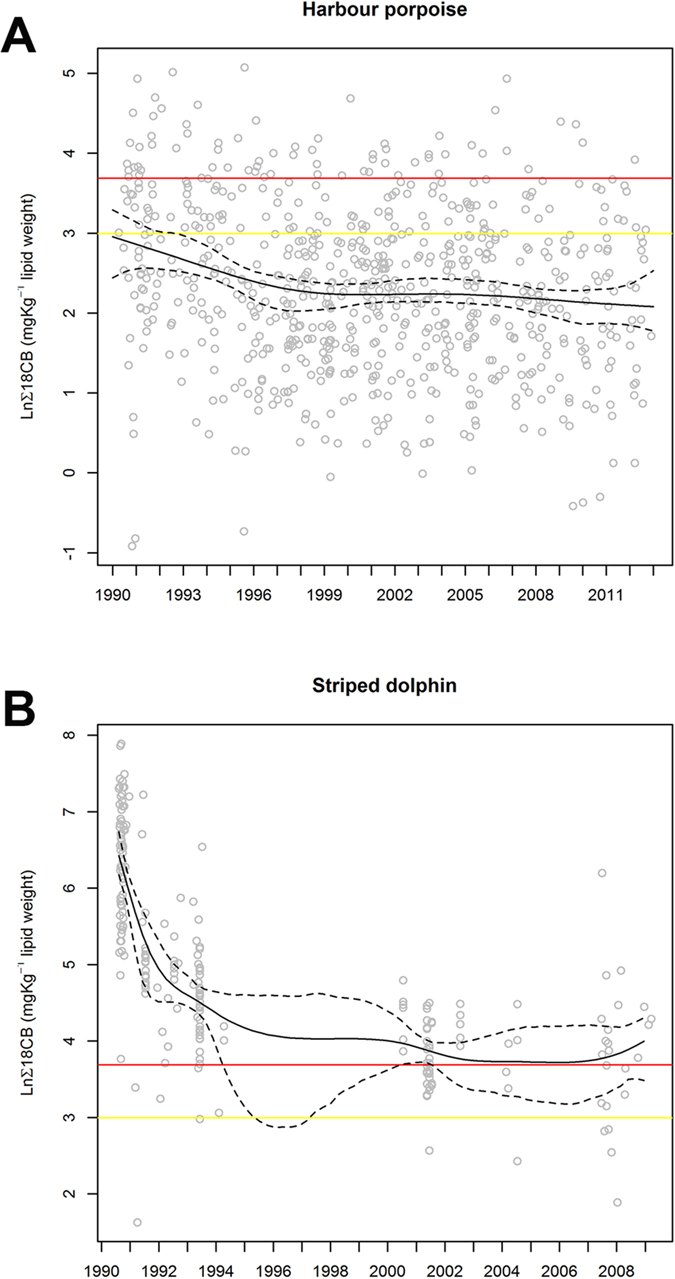
Temporal trends in ∑PCBs in UK-stranded harbour porpoise (*Phocoena phocoena*) and striped dolphins (*Stenella coeruleoalba*) in the western Mediterranean Sea. To assess the ∑PCBs trend we have fitted a Generalised Additive Model (GAM) to the data using the R (R Development Core Team, 2013) package *mgcv*. We used thin plate regression splines to do the smoothing and the degree of smoothing was determined by generalised cross validation. **(A)** Ln ∑PCBs (sum 18–25CB) mg/kg lipid concentrations in UK harbour porpoise blubber against date for all data for 1990–2012 (n = 706). The continuous line represents the smoothed trend from a Generalized Additive Model fitted to the data. The trend is statistically significant (p < 0.001, F = 11.76, residual df = 701.97, trend df = 3.03) against the null hypothesis of no trend. The dashed lines represent the 95% bootstrapped Confidence Intervals. The yellow line represents ln ∑PCBs equivalent to 20.0 mg/kg lipid and the red line 40 mg/kg lipid. **(B)** Ln ∑PCBs (sum 18–25CB) lipid concentrations in biopsied striped dolphin blubber from the Mediterranean Sea against date for all data for 1990–2009 (n = 220). Figure shows the natural logs (ln) of the whole data set plotted against the date found. The continuous line represents the smoothed trend from a Generalized Additive Model fitted to the data. The trend is statistically significant (p < 0.001, F = 55.45, residual df = 212.03, trend df = 6.97) against the null hypothesis of no trend. The dashed lines represent the 95% bootstrapped Confidence Intervals. The yellow line represents ln ∑PCBs equivalent to 20.0 mg/kg lipid and the red line 40 mg/kg lipid.

The temporal trend in ln ∑PCB lipid concentrations in UK HPs (1990–2012) and SDs from the western Mediterranean Sea (1990–2009) is shown in Fig. 1A,B. The temporal trend in both populations was highly statistically significant (p < 0.001). In UK-stranded HPs, ln ∑PCB concentrations declined slowly from 1990 to 1998 and then remained relatively stable from 1998 to 2012 against the null hypothesis of no trend (p < 0.001, F = 11.76, residual df = 701.97, trend df = 3.03) (Fig. 1A). In Mediterranean SDs, ln ∑PCB concentrations showed a marked decline from an initial peak in 1990, but then stabilized from 2003 to 2008, but with blubber ∑PCB concentrations still consistently exceeding all mammalian toxicity thresholds. The trend is statistically significant (p < 0.001, F = 55.45, residual df = 212.03, trend df = 6.97) against the null hypothesis of no trend (Fig. 1B).

**Figure 2:**
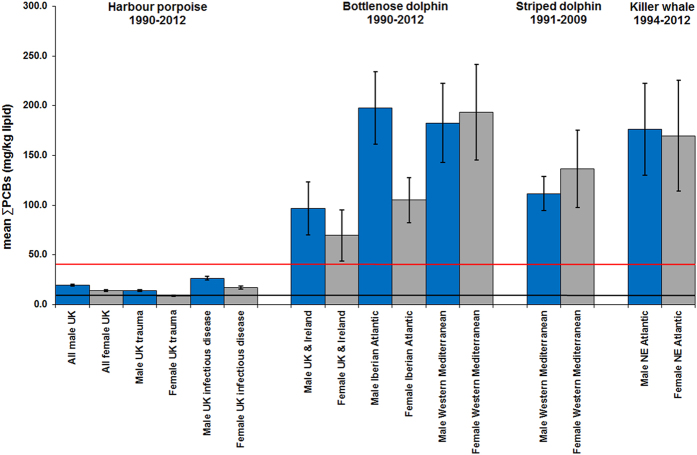
Mean ∑PCBs concentrations in male and female cetaceans (four species; all ages) The blue bars are males and the grey bars are females. The lower line is the equivalent ∑PCBs concentrations threshold (9.0 mg/kg lipid) for onset of physiological effects in experimental marine mammal studies^6^. The upper line is the equivalent ∑PCBs concentrations threshold (41.0 mg/kg lipid) for the highest PCB toxicity threshold published for marine mammals based on marked reproductive impairment in ringed seals in the Baltic Sea^6^. Mean ∑PCBs concentrations in male (n = 388) and female (n = 318) UK-stranded harbour porpoises (HPs) in 1990–2012. Mean blubber ∑PCBs (mg/kg lipid) concentrations in subsets of male (n = 201) and female (n = 144) UK-stranded HPs that died of acute physical trauma and male (n = 120) and female (n = 132) HPs that died of infectious disease from the same 1990–2012 period. Mean blubber ∑PCBs (mg/kg lipid) concentrations (1990–2012) shown for stranded/biopsied male (n = 29) and female (n = 17) bottlenose dolphins (BNDs) from UK and Ireland; male (n = 28) and female (n = 24) BNDs from Atlantic coast of Spain and Portugal and male (n = 9) and female (n = 11) BNDs from western Mediterranean Sea. Male (n = 50) and female (n = 39) striped dolphins from western Mediterranean Sea (1991–2009) and male (n = 5) and female (n = 19) KWs from NE Atlantic (1994–2012). Error bars = 1Standard Error of the Mean (SEM).

**Figure 3:**
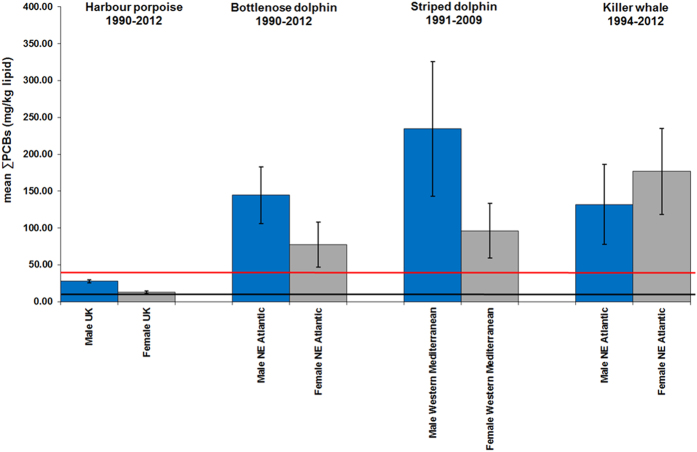
Mean ∑PCBs concentrations in male and female cetaceans (four species; adults only). The blue bars are adult males and the grey bars are adult females. The lower line is the equivalent ∑PCBs concentrations threshold (9.0 mg/kg lipid) for onset of physiological effects in experimental marine mammal studies^6^. The upper line is the equivalent ∑PCBs concentrations threshold (41.0 mg/kg lipid) for the highest PCB toxicity threshold published for marine mammals based on marked reproductive impairment in ringed seals in the Baltic Sea^6^. Mean ∑PCBs concentrations (mg/kg lipid) in adult male (n = 146) and female (n = 134) UK-stranded harbour porpoises in 1990–2012. Mean blubber ∑PCBs (mg/kg lipid) concentrations (1990–2012) shown for stranded/biopsied adult male (n = 20) and female (n = 14) bottlenose dolphins from UK and Ireland and the Atlantic coast of Spain and Portugal (NE Atlantic). Mean blubber ∑PCBs (mg/kg lipid) concentrations shown for adult male (n = 8) and female (n = 14) striped dolphins from the western Mediterranean Sea (1991–2009). Finally, mean blubber ∑PCBs (mg/kg lipid) concentrations shown for adult male (n = 3) and female (n = 18) killer whales from the NE Atlantic (1994–2012). Error bars = 1SEM.

**Figure 4:**
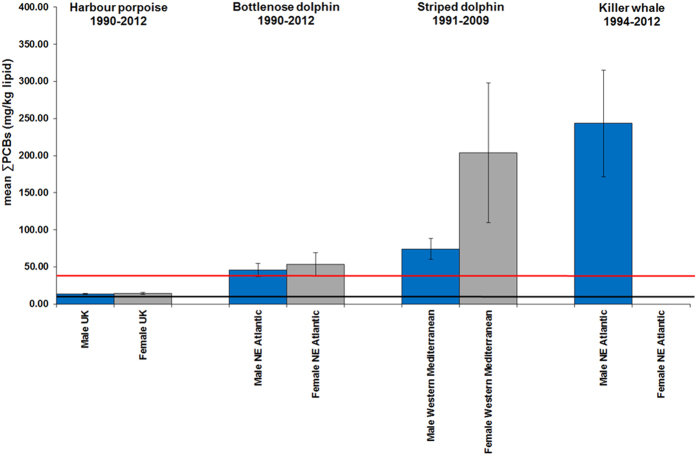
Mean ∑PCBs concentrations in male and female cetaceans (four species; juveniles only). The blue bars are juvenile males and the grey bars are juvenile females. The lower line is the equivalent ∑PCBs concentrations threshold (9.0 mg/kg lipid) for onset of physiological effects in experimental marine mammal studies^6^. The upper line is the equivalent ∑PCBs concentrations threshold (41.0 mg/kg lipid) for the highest PCB toxicity threshold published for marine mammals based on marked reproductive impairment in ringed seals in the Baltic Sea^6^. Mean ∑PCBs concentrations (mg/kg lipid) in juvenile male (n = 233) and female (n = 180) UK-stranded harbour porpoises (HPs) in 1990–2012. Mean blubber ∑PCBs (mg/kg lipid) concentrations (1990–2012) shown for stranded/biopsied juvenile male (n = 20) and female (n = 14) bottlenose dolphins from UK and Ireland and the Atlantic coast of Spain and Portugal (NE Atlantic). Mean blubber ∑PCBs (mg/kg lipid) concentrations shown for juvenile male (n = 17) and female (n = 15) striped dolphins from western Mediterranean Sea (1991–2009). Finally, mean blubber ∑PCBs (mg/kg lipid) concentrations shown for juvenile male (n = 2) and female (n = 0) killer whales from NE Atlantic (1994–2012). Error bars = 1SEM.

**Figure 5:**
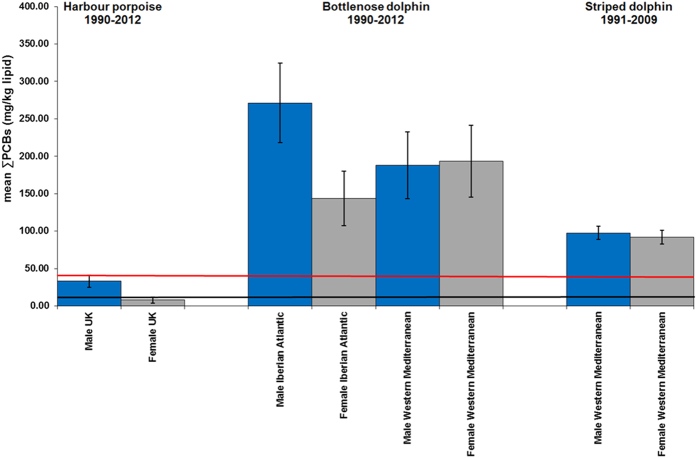
Mean ∑PCBs concentrations in male and female cetaceans (three species; sexual maturity unknown). The blue bars are males and the grey bars are females. The lower line is the equivalent ∑PCBs concentrations threshold (9.0 mg/kg lipid) for onset of physiological effects in experimental marine mammal studies^6^. The upper line is the equivalent ∑PCBs concentrations threshold (41.0 mg/kg lipid) for the highest PCB toxicity threshold published for marine mammals based on marked reproductive impairment in ringed seals in the Baltic Sea^6^. Mean ∑PCBs concentrations (mg/kg lipid) in male (n = 9) and female (n = 4) UK-stranded harbour porpoises of unknown sexual maturity (1990–2012). Mean blubber ∑PCBs (mg/kg lipid) concentrations (1990–2012) shown for stranded/biopsied male (n = 16) and female (n = 13) bottlenose dolphins (BNDs) from the Atlantic coast of Spain and Portugal and male (n = 8) and female (n = 11) BNDs from the western Mediterranean Sea (all sexual maturity unknown). Mean blubber ∑PCBs (mg/kg lipid) concentrations shown for male (n = 25) and female (n = 10) striped dolphins from western Mediterranean Sea (1991–2009) (sexual maturity unknown). Error bars = 1SEM.

**Figure 6:**
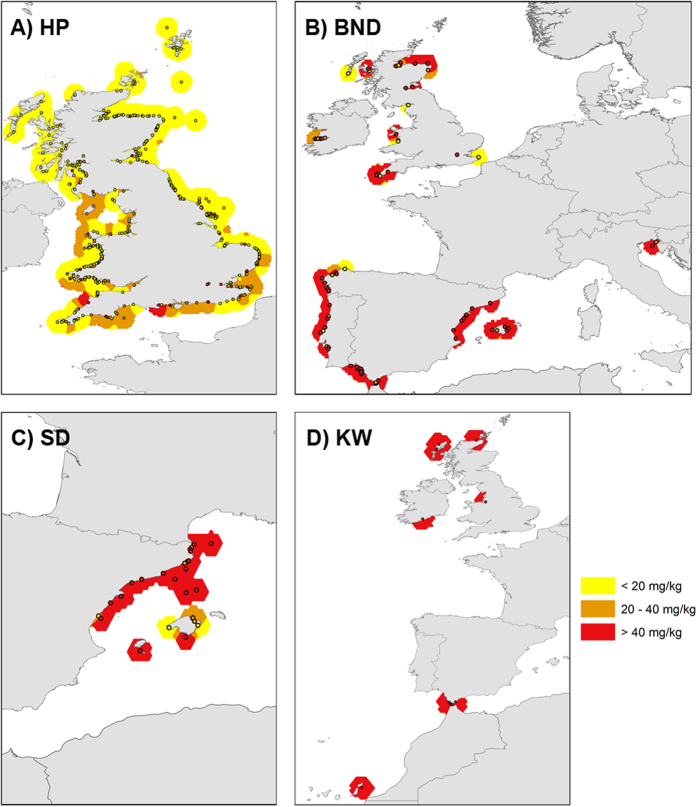
(**A–D**) Distribution map (smooth mean density kernel plots) of ∑PCBs data points in Europe – all cetacean species (all ages) from 1996–2012. **(A)** – HPs (n = 548); **(B)** – BNDs (n = 110); **(C)** – SDs (n = 71) and **(D)** – KWs (n = 21). Spatial distribution of ∑PCB lipid concentrations produced in *Esri ArcMap 10.1* (www.esri.com). Maps are displayed in the WGS84 co-ordinate system. Data points are shown along with local averages. These averages were calculated by kernel smoothing using a polynomial order 5 kernel with power = 0, ridge parameter = 50 and bandwidth based on the spatial distribution of the observations for each species: bottlenose dolphin 0.75 degrees; harbour porpoise 0.5 degrees; killer whale 1.2 degrees; striped dolphin 0.5 degrees. Both the data points and the local averages are displayed in three colours: yellow (∑PCB concentration =  < 20 mg/kg); orange (∑PCB concentration = 20–40 mg/kg lw); and red (∑PCB concentration =  > 40 mg/kg lw).

**Figure 7:**
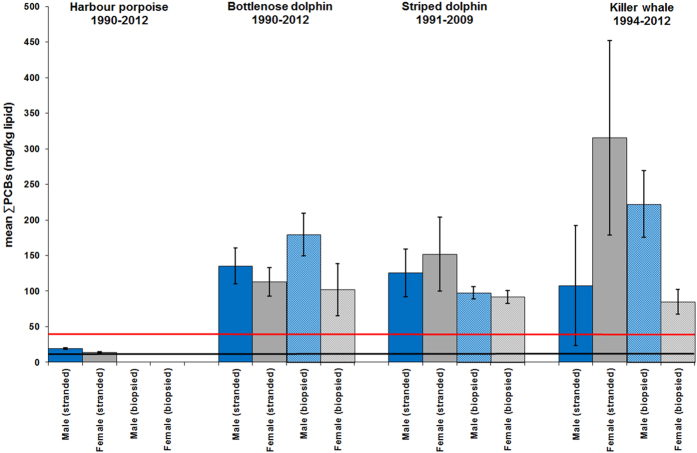
Mean ∑PCB concentrations in stranded and biopsied BNDs (1990–2012), SDs (1991–2009) and KWs (1994–2012) – all cetacean species (all ages). The blue bars are males and the grey bars are females. The lower line is the equivalent ∑PCBs concentrations threshold (9.0 mg/kg lipid) for onset of physiological effects in experimental marine mammal studies^6^. The upper line is the equivalent ∑PCBs concentrations threshold (41.0 mg/kg lipid) for the highest PCB toxicity threshold published for marine mammals based on marked reproductive impairment in ringed seals in the Baltic Sea^6^. Error bars = 1SEM.

**Figure 8:**
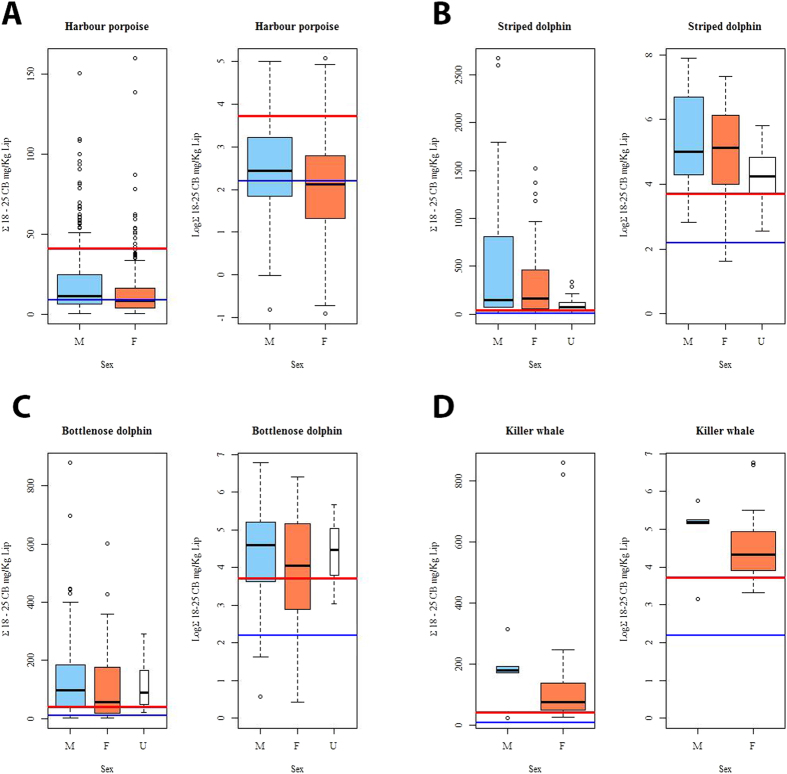
(**A–D**) Box and whisker plots for male (M) v female (F) v unknown sex (U) were generated for ∑PCB and ln ∑PCB concentrations for (**A**) all stranded HPs (1990–2012); (**B**) stranded or biopsied SDs (1990–2008); (**C**) stranded or biopsied BNDs (1990–2012); and (**D**) stranded or biopsied KWs (1994–2012). The lower line (blue) is the lower PCB toxicity threshold (=9.0 mg/kg lipid, as ∑PCB) for onset of physiological effects in experimental marine mammal studies^6^. The upper line is the highest PCB toxicity threshold (=41.0 mg/kg lipid, as ∑PCB) published for marine mammals based on marked reproductive impairment in ringed seals in the Baltic Sea^6^.

Some geographical regions in this study were global PCB “hotspots” for cetaceans. Mean ∑PCB lipid concentrations in BNDs and KWs from the NE Atlantic and in BNDs and SDs from the Mediterranean were among the highest recorded in cetaceans globally^9^, with males and females markedly exceeding all known PCB toxicity thresholds for marine mammals (Figs 2, 3, 4, 5). Results of kernel smoothing estimates show very high risk of PCB toxicity for KWs in NE Atlantic, BNDs off SW Iberia and in the northern Adriatic Sea and BNDs/SDs in the western Mediterranean (Fig. 6). ∑PCB data from biopsies of 152 free-living individuals constituted 38/131 (29.0%) of all BNDs; 99/220 (45.0%) of the SDs; and 15/24 (62.5%) of the KWs in this study. All HPs in the UK were stranded. Although biases can potentially occur with opportunistically sampled stranded cetaceans, the study population used here consisted of both compromised diseased individuals as well as healthy trauma cases, and no clear differences between mean ∑PCB concentrations in stranded and biopsied BNDs (1990–2012), SDs (1991–2009) and KWs (1994–2012) were observed (Fig. 7). More specifically, mean ∑PCB concentrations in stranded male and female SDs were slightly higher than biopsied male and female SDs but biopsied male BNDs had slightly higher ∑PCB concentrations than stranded male BNDs. For KWs, biopsied males and stranded females had higher ∑PCB concentrations than stranded male and biopsied female KWs, respectively (Fig. 7). Box and whisker plots (male v female) were generated for ∑PCB and ln ∑PCB concentrations in all stranded and biopsied HPs (1990–2012), BNDs (1990–2012), SDs (1990–2008) and KWs (1994–2012) (Fig. 8A–D).

We compared ∑PCB concentrations among regions within the NE Atlantic and Mediterranean Sea. Since our ∑PCB data is lognormal, we are able to use the sample geometric mean to estimate the population median (Tables 1 and 2), and that this estimator is unbiased for doing this^10^. Mean ∑PCB concentrations and geometric means (with upper and lower 95% confidence intervals) were generated for males and females of each cetacean species and region for all available data (n = 1,009) (Table 1) and for adults only (n = 401) (Table 2). These tabulated data were presented along with minimum and maximum values (i.e. range) for males and females for each species and region (Tables 1 and 2). In general, the ∑PCB sample geometric means (as estimator of population median values) were 25–65% lower than the (arithmetic) means for the same species, but still exceeded even the very highest marine mammal PCB toxicity threshold (∑PCB = 41 mg/kg lipid) for some BND, SD and KW groups in both NE Atlantic and Mediterranean Sea regions (Tables 1 and 2).

For males and females (all ages), high mean ∑PCB concentrations (>100.0g/kg lw) were found for BNDs off Iberian Peninsula (NE Atlantic), western Mediterranean and northern Adriatic Sea; for SDs in western Mediterranean and for KWs (NE Atlantic) (Fig. 2). Among adults, high individual female ∑PCB concentrations (>100mg/kg lw) were found in KWs from all NE Atlantic regions, in BNDs from England and Wales, and in SDs from the western Mediterranean Sea (Table 2). Mean ∑PCB concentrations in adult female KWs (176.8 mg/kg lipid) in the NE Atlantic greatly exceeded mean concentrations in adult female southern resident (55.4 mg/kg lipid) and transient (58.8 mg/kg lipid) KWs^10^ off British Columbia (NE Pacific). Adult female KWs in the UK and Ireland had the highest mean ∑PCB concentrations (224.8 mg/kg lipid) followed by KWs in the Strait of Gibraltar (215.4 mg/kg lipid). Mean ∑PCB concentrations in adult female BNDs (61.8–158.3 mg/kg lipid) and SDs (84.5–523.7 mg/kg lipid) in some western European populations also markedly exceeded those in southern resident and transient KWs off British Columbia^10^ (Fig. 3)(Table 2). Mean ∑PCB concentrations greatly exceeded all marine mammal PCB toxicity thresholds in mature (Fig. 3) and immature (Fig. 4) BNDs, SDs and KWs. Individuals for which the maturity status could not be established, mean ∑PCB concentrations still exceeded all marine mammal toxicity thresholds in BNDs and SDs (Fig. 5).

The toxicological data show that these populations greatly exceed concentrations at which severe toxic effects are known to occur. Pathological findings on necropsy consistent with immunosuppression and increased susceptibility to disease included macro-parasitic and bacterial pneumonias, high lung and gastric macro-parasite burdens, and generalised bacterial infections (septicaemias). These were regularly found on necropsy in stranded HPs^5^, BNDs and KWs. In Mediterranean SDs distemper due to cetacean morbillivirus (CeMV) infection^9^ was frequently seen. Several stranded KWs also had multiple dental infections leading to large mandibular abscesses identified on necropsy^12^. High ∑PCB contamination can cause immunosuppression^7^ and may be a significant contributing factor in the death of many of the stranded individuals that had fatal infectious diseases on necropsy.

For necropsied cetaceans stranded in the UK (1990–2012), ∑PCB data was obtained for 706 HPs, 38 BNDs and 7 KWs (Tables 1 and 2). For BNDs (UK), causes of death included trauma (n = 5 male; n = 2 female); infectious disease (n = 2 male; n = 4 female). For the UK-stranded KWs, causes of death included starvation (n = 2), infectious disease (n = 2) and live stranding (n = 2). A cause of death could not be determined for 25 HPs, 11 BNDs and 1 KWs. ∑PCB lipid concentration in UK-stranded male HPs of all ages from 1990–2012 was significantly higher (sample mean = 19.4 mg/kg lw; sample median = 11.5 mg/kg lw; n = 388) than for females of all ages (sample mean = 13.8 mg/kg lw; sample median 8.35 mg/kg lw; n = 318) (ANOVA, P < 0.001) (Fig. 2).

∑PCB lipid concentrations in subgroups of “healthy” male UK-stranded HPs that were generally in good nutritive condition and died of acute physical trauma (sample mean = 13.8 mg/kg lw; sample median = 9.57 lw; n = 201) had significantly lower ∑PCB than male HPs that were in poorer condition and died of a range of infectious diseases (sample mean = 26.5 mg/kg lw; sample median = 18.9 mg/kg lw; n = 120) (ANOVA, P < 0.001). In female UK-stranded HPs that died of acute physical trauma, ∑PCB lipid concentrations (sample mean = 8.50 mg/kg lw; sample median = 6.41 mg/kg lw; n = 144) were significantly lower than female HPs that died due to a range of infectious diseases (sample mean = 16.8 mg/kg lw; sample median = 12.2 mg/kg lw; n = 132) from the same period (ANOVA, P < 0.001) (Fig. 2). Male and female HPs that died of starvation were all in a poor or emaciated condition. Both male HP starvation cases (sample mean = 20.7 mg/kg lw; sample median = 12.5 mg/kg lw; n = 26) and female HP starvation cases (sample mean = 27.9 mg/kg lipid; sample median = 9.74 mg/kg lw; n = 16) had higher ∑PCB concentrations than the physical trauma group. For UK-stranded HPs (both sexes), mean ∑PCB mg/kg lipid was 150–328% greater in infectious disease or starvation cases as compared to trauma cases. The increase in ∑PCB on a sample median basis was 130–198% greater for HP disease or starvation cases as compared to HP trauma cases.

### Population Dynamics

Such sustained and elevated PCB burdens are likely to have significant effects at the population level in European BNDs, SDs and KWs. Although we cannot directly and causally link high ∑PCB exposures to cetacean population declines, many of these populations are either very small, or show evidence of major and long-term declines or a significant contraction of range. Only very small KW populations are now found in industrialised regions of Europe^13^,^14^,^15^. There is only one resident population in southern Europe^14^: a tuna-feeding KW subpopulation in the strait of Gibraltar comprising two pods totalling 36 individuals^13^. These animals have been monitored every year since 1999. Only six mature females were reported to have calved between 1999 and 2011 and these produced only five calves living more than 1 year. This 6.4% annual female fecundity rate over a 13-year period is one of the lowest recorded reproductive rates for KWs globally and the subpopulation was recommended for listing as “Critically Endangered” due to small population size^14^. Around NW Scotland and Western Ireland, a small group of only nine KWs is regularly seen^15^. This group of marine-mammal-eating KWs comprises four adult males, two adult females and three adult female or sub-adult males. Although studied over a 19-year period, no calves have ever been reported within this group^15^. No other resident or coastal KW groups are found off the Iberian Peninsula or in the Bay of Biscay, although occasional offshore KW sightings occur (e.g.^16^). Long-term cetacean stranding records in the Netherlands show that KWs were first recorded on the Dutch North Sea coastline in 1783, observed regularly from 1918 to 1963 and completely ceased thereafter until two single KW strandings in 2009 and 2010^17^.

Historic strandings data suggest that multiple BND resident or coastal groups in Europe became depleted or locally extinct in the late-1960s to mid-1970s, including those in the UK (e.g. Morecambe Bay; East coast of England)^18^ and the North Sea Dutch coast^19^. The last member of a resident BND population at Arcachon, France, died in 2003^20^ and the small resident BND group (current census n = 27) in the Sado Estuary, Portugal, showed decline due to low calf survival over several decades^21^. Blubber PCB levels for two females and one male BND (sampled between 1995 and 1997) from this population ranged from 37.1 to 114.0 mg/kg lw. The Mediterranean Sea remains a global PCB hotspot^1^ where most of the extant marine mammal species, including SDs, BNDs and short-beaked common dolphins (CDs)(*Delphinus delphis*) have declined over decades^14^,^22^. The current IUCN Red List conservation status of BNDs and SDs in the Mediterranean Sea is “Vulnerable”. The Mediterranean CD subpopulation is now classified as “Endangered” after experiencing major and generalized decline over the last 40–50 years, particularly in the northern Adriatic Sea and in the eastern Ionian Sea^8^. Blubber PCB concentrations are significantly higher in CDs and SDs in the Mediterranean Sea compared to the much more abundant NE Atlantic populations^23^.

For HPs in the NE Atlantic, a single continuous population ranges from France to northern Norway. A separate much smaller Iberian population is estimated to comprise only 4,398 (CV = 0.92) porpoises^24^. In the Baltic Sea, two separate HP subpopulations exist: one in the western waters (Kattegat, the Belt Seas) estimated at 40,475 individuals (CV = 0.235) and listed as “Vulnerable” (by HELCOM)^25^, and another in the Baltic Sea proper, which is now very small and has suffered decades of decline and is listed as “Critically Endangered”^8^. A history of hunting, high chemical pollutant exposure and accidental entrapment in commercial fishing gear (bycatch) are thought to have been responsible for this decline^24^. A total of 375,358 (95% CI = 256,304–549,713) porpoises was estimated for EU Atlantic continental shelf waters including the Iberian and western Baltic (sub-)populations (July 2005)^16^. For the NE Atlantic (UK) HP population, a longer calving interval, lower pregnancy rate and later maturation and higher rates of reproductive abnormalities were also recently identified in a necropsy study of 329 female UK-stranded HPs (1990–2012), as compared to HP populations in much less PCB-polluted regions like Iceland and the Gulf of Maine/Bay of Fundy (NW Atlantic)^26^. Direct observations of reproductive failure (foetal death, abortion, dystocia or stillbirth) were observed in 25/127 (19.7%) of necropsied mature female HPs in the same study^26^.

### Consideration of alternative causes of mortality

Accidental entrapment in commercial fishing gear (bycatch) is often considered to be the greatest conservation threat globally to small cetaceans^8^,^27^. In UK strandings and necropsy data (1990–2013 inclusive) bycatch was diagnosed as the cause of death for 296/615 (48.1%) of UK-stranded common dolphins (*Delphinus delphis*) (CDs) and 338/1983 (17.0%) of HPs. In contrast, only 9/130 (6.9%) UK-stranded SDs; 4/71 (5.6%) BNDs and 0/7 (0.0%) KWs were diagnosed as bycatch during the same period. Bycatches recorded under the 2013 UK dedicated bycatch sampling programme included 101 days on pelagic trawls and 346 days on static gear vessels and included observed bycatches of 18 HPs, 11 CDs, two SDs and a single BND. Six common dolphins were recorded in pelagic trawls. All other cetacean bycatches were recorded from static net fisheries. Preliminary bycatch estimates for the entire UK fleet during 2013 from systematic observer-based studies provided estimates of HP bycatch of around 1917 animals assuming no pingers (acoustic bycatch deterrent devices) were used (CV = 0.126) and 1652 animals if all UK boats over 12 m used pingers correctly (under *EU regulation 812*)(CV = 0.147)^28^. Other estimates for the entire UK fleet in 2013 indicate around 320 CDs and around 470 seals were bycaught^28^. Bycatch of BNDs, however, was considered too rare to estimate for the entire UK fleet and KW bycatch was not recorded^28^. A bycatch observer study for the Irish albacore tuna (*Thunnus alalunga*) drift-net fishery undertaken in offshore waters of the NE Atlantic in the 1990s found the highest bycatch rates in CDs followed by SDs, with much lower numbers of bycaught BNDs and no KW bycatch^29^. The highest bycatch rates generally occur in the most numerically abundant species in the NE Atlantic region, HPs and CDs, but with much lower rates of bycatch in BNDs and bycatch unrecorded in KWs in recent years^30^. Very low numbers of bycatches were found in the long-term observer-based studies of the resident European BND populations:- BNDs in Shannon estuary, Ireland (population size = 107–140; zero bycatch recorded since 1993)[S. Berrow, unpublished data]; Sado estuary, Portugal (population size = 27; zero bycatch recorded from 1983–2013) (Manuel E. Dos Santos, *pers. com.*) and Slovenia, northern Adriatic Sea (population size estimate = 74; 95% CI = 57–90; 2 cases of bycatch since 2002) [T. Genov, unpublished data, see also^31^]. Collectively, these necropsy and fisheries bycatch observer studies from approximately 1990 onwards indicate that bycatch is unlikely to be driving population declines of BNDs or KWs around the UK or in the wider NE Atlantic during this period.

Other potential causes of cetacean mortality include ship-strike; acoustic disturbance from high-intensity/anthropogenic acoustic sources; nutritional limitation caused by reduced prey availability (e.g. overfishing or climate change); biotoxins (harmful algal blooms) and infectious disease (e.g. cetacean morbillivirus, CeMV)^32^. Using CSIP UK strandings and necropsy data (1990–2013 inclusive) ship-strike was a relatively infrequent cause of death accounting for only 17/1983 (0.8%) stranded HPs; 0/130 (0.0%) SDs; 0/71 (0.0%) BNDs and 0/7 (0.0%) KWs necropsied in the UK. All tests for biotoxins in UK-stranded cetacean tissues were negative (or at trace levels) since 1990^32^,^33^.

The potential impacts of anthropogenic high-intensity acoustic sources on cetaceans include mass stranding events (MSEs) that have occurred on a global basis, predominantly involving beaked whales exposed to mid-frequency active sonars in naval exercises^34^,^35^,^36^. Non-beaked whale MSEs with a probable acoustic cause have occurred, such as a 2008 CD MSE in the UK, but are very rare in European waters^32^. No acoustically-induced MSEs were recorded in HPs, SDs, BNDs or KWs in European waters. Acoustically-induced cetacean MSEs have been recorded in the Mediterranean Sea but also primarily involved beaked whales^36^,^37^.

In the Mediterranean and Black Seas, all cetacean species where population assessments have occurred are considered to be declining^8^,^14^,^22^. A range of threats has been proposed including hunting (historically); bycatch; overfishing/prey depletion; habitat degradation; morbillivirus infection and chemical and acoustic pollution^8^,^14^,^22^. Empirical evidence for the effects of prey depletion (e.g. due to overfishing or climate change effects) leading to nutritional limitation is limited for cetaceans, but diet/nutrition effects have been linked to recruitment in KWs and fin whales (*Balaenoptera physalus*) [reviewed in^26^]. Such processes would potentially increase PCB toxicity in malnourished animals by mobilising lipophilic PCBs from blubber to other body compartments^7^,^23^. A recent global review of Cetacean Morbillivirus (CeMV) found that SDs have been negatively impacted by CeMV epizootics in 1990–1992 and 2006–2008^38^ and probably again in 2011 in the western Mediterranean^39^. The largest CeMV epizootic in 1990–1992 was associated with very high PCB concentrations^9^. A CeMV epizootic also occurred in long-finned pilot whales (*Globicephala melas*) in the Mediterranean Sea in 2006–2007^40^. In contrast, only small periodic CeMV-related mortalities have occurred in individual cetaceans in NE Atlantic region^38^. No large scale epizootics of CeMV^38^ or of any other pathogens are known to have occurred in any cetacean species in NE Atlantic region since 1990.

## Discussion

The stabilised temporal trends in blubber PCB concentrations in cetaceans in Europe contrast with other industrialised northern hemisphere regions, such as North America, where PCBs have continued to decline in BND^41^ and KW^11^,^23^ populations. PCBs were banned in closed systems in the USA in 1979^1^ and in 1981 in the UK^4^, but were not phased out until 1987 in European countries that border the Mediterranean Sea^1^,^42^. Mean ∑PCB concentrations in adult female KWs in our study substantially exceeded concentrations in both southern resident and transient female KWs off British Columbia^11^,^23^. Where females are reproducing normally, blubber PCB concentrations are known to fall significantly with successive pregnancies due to maternal offloading through gestation and lactation^7^,^41^,^42^. The relatively high ∑PCB concentrations in many adult female KWs in this study is consistent with reproductive failure in at least some individuals, due to either adult females failing to offload PCBs to calves during pregnancy and lactation^7^ or re-accumulation of dietary PCBs in some post-parturient and reproductively senescent KWs^11^.

Mean blubber ∑PCB concentrations in male KWs in this study were higher than male “southern resident” KWs off British Columbia (NE Pacific) in the 1990s and were only slightly lower than male “transient” (marine mammal eating) KWs from the same region^11^,^41^. Very high mean PCB concentrations have been recorded in blubber biopsies of adult male (n = 4) marine mammal-eating West Coast “transient” KW ecotype off California, USA (mean ∑PCB = 630 mg/kg lipid)^43^; in individual stranded KWs in Oregon State^23^; and in a transient adult female that stranded in Washington State in 2002 with ∑PCB = 1,100 mg/kg lipid^44^. For BNDs, only a population in a highly polluted Superfund site in Brunswick, Georgia (USA) exposed to a major point source of PCB pollution with *Aroclor 1268*^45^ had mean exposures similar to many BND groups in our study. HP blubber ∑PCB concentrations in this study were similar to those associated with immunosuppression and infectious disease mortality in previous multivariate studies of UK-stranded HPs (statistically independent of age, sex, sexual maturity and two quantitative indices of nutritional status)^5^ and similar to those associated with impaired reproduction in adult female HPs in UK waters^26^.

A large number of factors can influence PCB exposure and subsequent PCB concentrations in blubber including, but not limited to, blubber sample condition, diet, degree of maternal offloading, year of sampling, season, age, gender, location, species, body condition, and whether or not an animal was sampled in good health^1^,^4^,^7^. Some of these we can control for statistically as potentially confounding factors, others we cannot. Nevertheless, for SDs, BNDs and KWs around the Iberian Peninsula (both NE Atlantic and Mediterranean regions), mean and median ∑PCB concentrations levels were among the highest recorded for cetaceans globally, regardless of sample type/nutritional condition/sex/year of sampling, etc. In terms of blubber sample quality, we are able to obtain 954/1081 ( = 88.3%) fresh or slightly decomposed stranded or biopsied blubber samples, effectively negating the potential bias of sample degradation on PCB determination. Nor did we see any significant overall differences in mean ∑PCB concentrations between stranded and biopsied BNDs, SDs or KWs. This shows that the ∑PCB concentrations in stranded animals were not fundamentally biased in comparison to the free-living biopsied cetaceans.

In relationship to nutritional status, we can show that both male and female HPs that died of trauma had the lowest ∑PCB concentrations and this increased by 190–198% for infectious disease cases and by 130–328% for starvation cases. Although we lack data to test this “nutritional loss” effect rigorously on ∑PCB concentrations in SDs, BNDs and KWs, we can show that the excessively high ∑PCB concentrations occurred in virtually all individual SDs and BNDs around the Iberian Peninsula and for virtually all individual KWs across the NE Atlantic region, irrespective of blubber sample quality, year of sampling, season, gender, nutritive condition and whether or not an animal was sampled in good health.

There are also marked species-specific variations in susceptibility to PCB exposure. The lower PCB toxicity threshold used in this study was predominantly derived from non-cetacean species such as seals, otters, and mink^7^. It is well recognized that mink are one of the most PCB-sensitive species and so it is highly possible that cetaceans are less sensitive to PCB exposure than species like mink. If so, the use of the Kannan threshold^7^ is likely to overestimate the true PCB risk to cetaceans. However, this lower PCB toxicity threshold is equivalent to only 9 mg/kg lipid (as ∑PCB) – whereas many BND, SD and KW groups in this study had one order of magnitude greater mean or median ∑PCB concentrations (50–350 mg/kg lipid). So, even if the lower PCB toxicity threshold does significantly over-estimate the likely toxicity of PCBs on cetaceans – the very high ∑PCB exposures recorded empirically in European cetaceans in this study (>1,000% greater than 9 mg/kg lipid) still provides compelling evidence for the inherent PCB toxicity risk in this study.

Current threats to cetaceans from persistent organic pollutants (POPs) in Europe appear to be restricted to PCBs. Marked and ongoing declines in tissue concentrations of organochlorine pesticides (e.g. DDTs) have occurred in UK-stranded HPs^4^ and in western Mediterranean SDs^3^ and BNDs^42^. Similar marked declines in tissue contaminants have occurred in UK HPs for butyltins^46^ and brominated flame retardants following a 2004 EU-ban^4^. Other newer chemical compounds including the alternative brominated and chlorinated flame retardants such as tetrabromo-p-xylene (TBX), tetrabromo-o-chlorotoluene (TBCT) and 2,3-dibromopropyl-2,4,6-tribromophenyl ether (TBP-DBPE) and Dechlorane Plus (DDC-CO) isomers^47^, perfluoroalkyl substances (PFASs) such as perfluorooctane sulphonate (PFOS) and perfluorooctanoic acid (PFOA) in^48^ and emerging organophosphorus flame retardants (PFRs) and plasticisers^49^ were detected in some UK-stranded HP samples, and with the possible exception of PFOS, were at either very low levels or occurred below levels of analytical detectability.

Many European seas (e.g. Baltic, Mediterranean and North Seas) are surrounded by highly industrialised land masses with high human population densities which may at least partly explain the high PCB exposures we have detected^1^. Mass production of PCBs commenced in 1929, reaching peak levels in the 1960s and 1970s, when many European cetacean populations already appear to be declining. Nearly 97% of the historical use of PCBs occurred in the northern hemisphere^50^, with the marine environment acting as the ultimate PCB “sink”. By the late 1990s, however, only 1% of globally manufactured PCBs were estimated to be in sea water, with 6–7% in marine sediments (reviewed in^1^), while as of 2005, 1.1 million tons of PCB contaminated material still required disposal by EU Member States, most notably France and Spain^51^. PCB effects are likely to be particularly pernicious in highly exposed European BND, SD and KW populations by markedly suppressing reproduction and probably subsequent recruitment at individual and population levels. EU regulations to mitigate PCB pollution currently appear insufficient to protect cetacean top predators in the NE Atlantic and Mediterranean Sea. As sentinels of marine ecosystems, monitoring PCB concentrations in marine mammals should be considered for inclusion as an indicator under descriptor 8 within the *European Marine Strategy Framework Directive* (MSFD) (Directive 2008/56/EC). Our results indicate that European marine mammal habitats are unlikely to meet Good Environmental Status within the MSFD by 2020, and that several dolphin species are unlikely to achieve Favourable Conservation Status now under *EC Habitats Directive* (Council Directive 92/43/EEC). Further steps to reduce PCB inputs into the European marine environment should include stricter controls on disposal of PCBs, e.g. in buildings with sealants containing PCBs^4^,^52^. Also, measures should be taken to limit PCB bioavailability to marine food webs, such as improved management of dredging of harbours/ports, as well as improved containment of PCB environmental contamination from industrial buildings/equipment and domestic waste disposal (e.g. landfill).

In conclusion, this pan-European meta-analysis of stranded or biopsied cetaceans demonstrates that several European cetacean species, specifically BNDs, SDs, and KWs, currently have markedly elevated blubber PCB concentrations. Particular “PCB hotspots” included the western (SDs and BNDs) and central (BNDs) Mediterranean Sea and SW Iberia, the Gulf of Cadiz (BNDs) and the Strait of Gibraltar (BNDs and KWs). Despite an EU ban on the use and manufacture of PCBs in the mid-1980s, blubber PCB concentrations are still very high, possibly having reached a “steady state” between environmental input and degradation, meaning that high PCB exposures are set to continue for the long-term in cetacean top predators in Europe. These high and stable PCB exposures are associated with small populations, long-term population declines or contraction of range in several dolphin species in Europe (NE Atlantic and Mediterranean Seas) that were not adequately explained by other factors (e.g. bycatch or other anthropogenic causes of mortality). Bycatch is common in the most abundant cetacean species in Europe, but is comparatively rare in BNDs and virtually unrecorded in recent years for KWs, suggesting that the ongoing population declines in these two species are predominantly driven by other processes, with bioaccumulation of PCBs through marine food chains being the predominant factor. A lack of recruitment in monitored KW and BND populations is also consistent with PCB toxicity as the likeliest cause of their declines. In the Mediterranean Sea, the SD has suffered recurrent CeMV mortalities, which may have been exacerbated by the high and immunotoxic level of PCB exposure. Without significant mitigation, PCBs will continue to drive population declines or suppress population recovery in Europe for many decades to come. Measures to significantly reduce inputs of PCBs into the marine environment from terrestrial and other sources are urgently needed. Further studies are also needed to better assess PCB exposure and quantify toxic effects in marine apex predator populations in Europe. Finally, the potential impact of PCB bioaccumulation in marine ecosystems may extend beyond European waters, particularly in globally distributed marine apex predators such as KWs, false killer whales (*Pseudorca crassidens*) and great white sharks (*Carcharodon carcharias*).

## Methods

Between January 1990 and December 2012, HPs (n = 706), SDs (n = 220); BNDs (n = 131) and KWs (n = 24) in NE Atlantic and Mediterranean Sea regions of Europe were necropsied (HP n = 706; SD n = 119; BND n = 93; KW n = 9) or biopsied (SD n = 101; BND n = 38; KW n = 15) for skin and blubber samples using standardised methodologies^5^,^9^.

### Necropsies of stranded cetaceans (n = 929)

A total of 929 cetaceans were sampled or necropsied after stranding on a beach or being found dead at sea. These comprised 706 HPs; 121 SDs 93 BNDs and 9 KWs. In the UK, stranded or bycaught HPs (n = 706), BNDs (n = 38) and KWs (n = 7) were necropsied using methods based on a standardised European necropsy protocol^53^. Necropsies were also conducted on stranded SDs (n = 119) from western Mediterranean, BNDs from Galicia, Spain (n = 11) and Portugal (n = 5) and a single KW stranded in the Strait of Gibraltar, Spain using similar methods. Wherever possible, stranded cetaceans in fresh or slight decomposition were prioritized for necropsy to limit decomposition of tissue samples including blubber. Fresh or slightly decomposed carcasses comprised 802/929 (86.3%) of all necropsies and included 660 HPs; 103 SDs; 33 BNDs and 6 KWs. Moderately decomposed carcasses accounted for 72/929 (7.75%) of necropsied carcasses including 37 HPs; 21 BNDs; 11 SDs; and 3 KWs. Only 18/929 (1.94%) carcasses were in advanced decomposition comprised 8 HPs; 5 BNDs and 5 SDs. State of decomposition could not be determined for 37/929 (3.98%) carcasses comprising 34 BNDs; 2 SDs and 1 HPs. Sex was determined on necropsy. Sex was not recorded or could not be determined (e.g. due to loss of gonads from scavenging animals) for 65 SDs and 6 BNDs. Causes of death in UK-stranded animals were determined by specific and established diagnostic criteria^5^. Additional chlorobiphenyl congener and other data were included from a single KW that stranded at Roches Point, Cork harbour, Ireland and was sampled on 8^th^ July 2001^12^.

### Blubber samples from biopsied cetaceans (n = 152)

Blubber biopsies of free-living SDs from the western Mediterranean Sea (n = 101), BNDs from the Gulf of Cadiz (n = 11), Strait of Gibraltar (n = 11) and northern Adriatic Sea (n = 6), and skin/blubber biopsies of KWs from Canary Islands (n = 8) and Strait of Gibraltar (n = 7) were obtained using a range of standardised techniques including use of a cross-bow^54^. For BNDs and SDs in the western Mediterranean Sea, the skin and blubber tissue biopsy was excised from bow-riding dolphins using a sterile biopsy dart of the butterfly valve type, shot with a spear gun or a compressed air pistol from a boat, a non-destructive technique commonly used in cetacean research^54^. Darts were aimed at the region posterior to the dorsal fin. The precise age or sex of the individual samples was unknown. The samples collected contained about 0.8 g of blubber. Once collected, the skin and blubber biopsy samples were wrapped in aluminum foil and preserved at −20 or −80C until analysis. Sex of biopsied animals was determined using molecular techniques^55^. Additional chlorobiphenyl congener and other data were included from BNDs (n = 8) biopsied in the Shannon estuary, Ireland in September 2000^56^.

### Assessment of sexual maturity in cetaceans

Sexual maturity was determined in necropsied animals by analysis of gonadal material^57^. For those individuals where sexual maturity status data based on gonadal assessments were unavailable, published data on age and length at both sexual and physical maturity were used as criteria for assessing maturity status – BNDs^58^, SDs^59^,^60^ and KWs^61^. Animals assessed as sexually immature comprised HPs (n = 413), SDs (n = 62), BNDs (n = 35) and KWs (n = 2). Animals assessed as sexually mature comprised HPs (n = 280), SDs (n = 59), BNDs (n = 41) and KWs (n = 21). Sexual maturity status could not be determined for HPs (n = 13), SDs (n = 99), BNDs (n = 55) and KWs (n = 1).

### Determination of ∑PCB in blubber samples (mg/kg lipid)

Two different laboratories conducted chemical determination of PCB concentrations in 1073/1081 (99.3%) blubber samples. The *Centre for Environment, Fisheries and Aquaculture Science (Cefas)* in Lowestoft, UK conducted analyses of ∑PCB for 706 HPs, 82 BNDs and 24 KWs stranded or biopsied in the NE Atlantic or central Mediterranean Sea between 1990 and 2012. The *Department of Animal Biology and Biodiversity Research Institute (IRBio), University of Barcelona*, in Barcelona, Spain analysed 220 SDs and 41 BNDs, predominantly from the western Mediterranean Sea. A third laboratory, the *Marine Institute*, in Galway, Ireland examined blubber biopsies from 8 BNDs from the Shannon estuary, Ireland^56^.

### *Cefas* data

The blubber samples at the *Cefas* lab were analysed for 25 individual chlorobiphenyl congeners (IUPAC numbers: 18, 28, 31, 44, 47, 49, 52, 66, 101, 105, 110, 118, 128, 138, 141, 149, 151, 153, 156, 158, 170, 180, 183, 187, 194) (sum25CBs mg/kg lipid) using internationally standardised methodology^4^,^5^. After thawing, the homogenised subsamples (~5g) were dried by mixing with anhydrous sodium sulphate and storing in a freezer for a minimum of 12 hours prior to further analysis. The samples were subjected to Soxhlet extraction using of acetone: *n*-hexane 1:1 (*v:v*) for 5.5 hours. The total extractable lipid content was determined gravimetrically after evaporation of the solvent from an aliquot of the uncleaned extract. Depending on the lipid content of the samples, varying volumes of the biota extracts were cleaned to have ~50 mg of lipid in the samples for PCB analysis. For marine mammal blubber samples, this was usually 1mL out of the 100mL extract.

For clean-up and PCB analyses, an aliquot of the blubber extracts were cleaned up and fractionated using alumina (5% deactivated) and silica (3% deactivated) columns, respectively. The final GC-ready fractions were spiked with PCB53 internal standard and made up to a final volume of 1 ml. PCB concentrations were determined with an Agilent 6890 GC with μECD. The separation of analytes was performed on a 50.0 m × 200 μm, 0.33-μm-film-thickness DB-5 capillary column (J&W). The carrier and ECD make-up gas were hydrogen (32.2 psi constant pressure, initial velocity 50 cm/s) and argon/methane (95:5), respectively. The initial oven temperature was 90 °C, held for 2.00min, then increased to 165 °C at 15 °C/min, to 285 °C at 2 °C/min, and finally held for 23 min. The injector temperature and detector temperature was 270 °C and 300 °C, respectively. A 1-μl extract was injected in splitless mode with a purge time of 2 min. The PCB standard solutions contained the following 27 compounds in iso-octane: Hexachlorobenzene; *p,p’*-DDE; CB101; CB105; CB110; CB118; CB128; CB138; CB141; CB149; CB151; CB153; CB156; CB158; CB170; CB18; CB180; CB183; CB187; CB194; CB28; CB31; CB44; CB47; CB49; CB52; CB66; together with the internal standard CB53. Quantitation was performed using internal standards and 7 calibration levels (range 0.5–100ng/ml).

For Quality Assurance and Quality Control the *Cefas* laboratory participates biannually in proficiency testing scheme Quasimeme (Quality Assurance of Information for Marine Environmental Monitoring in Europe) as external quality assurance. All analyses were carried out under full analytical quality control procedures that included the analysis of certified reference material(s) and a blank sample with every batch of 10 samples analysed so that the day-to-day performance of the methods could be assessed. If levels of target analytes in the samples were outside of the range of the instrument calibration, extracts were diluted to be within range and re-analysed. Reference material used was BCR349 (cod liver oil; European Bureau of Community reference), which has been used in the Cefas lab since 1993. The results obtained for the reference materials were plotted as Shewhart quality control charts for each compound determined. The charts had previously been created by the repeated analysis of the above certified reference materials in the *Cefas* Lowestoft Laboratory using the North West Analytical Quality Analyst software™ (Northwest Analytical Inc., USA). Warning and control limits had been defined for the charts as 2σ and 3σ – 2x and 3x the standard deviation from the mean for each compound. The results obtained for all samples analysed were accepted as valid as the results for the certified reference materials were within the limits set by the control charts.

### *IRBio* data

Lipid weight concentrations (mg/kg lipid) of 23 individual chlorobiphenyl congeners (IUPAC numbers: 95, 101, 136, 110, 151, 144, 149, 118, 153, 141, 138, 187, 183, 128, 174, 177, 202, 171, 180, 170, 201, 203 and 195) (sum23CBs mg/kg lipid) were generated for 220 SDs and 41 BNDs from western Mediterranean Sea using internationally standardised methodology^42^,^62^. Approximately 1 g of tissue was ground with anhydrous sodium sulphate using a mortar. The mixture was extracted with n-hexane for 4 h in a Soxhlet apparatus with a capacity of 125 ml. The solution obtained was concentrated to 40 ml. A portion of this extract (10 ml) was used to gravimetrically determine the quantity of extractable fat per gram of blubber. A further quantity was mixed with sulphuric acid for the purpose of cleaning it, and the resulting extract was concentrated to 1 ml and centrifuged for five minutes.

A sample volume of 0.5 μL was injected in splitless mode (1 min splitless vent time) into a Thermo Scientific ITQ 900 GC/MS system equipped with a 30-m DB-5 MS (95% metylsilicone and 5% phenyl) fused-silica capillary column with an ID of 0.25 mm and a film thickness of 0.25 μm, with helium as the carrier gas at constant flow of 1 ml min^−1^. Injection port and transfer line temperatures were 270 and 300 °C, respectively. The oven temperature program was as follows: 60 °C for 1 min, an increase to 315 °C at 6 °C min^−1^, and a final hold of 20 min. Source temperature was 200 °C. Mass spectrometry was performed in electron impact mode. Full-scan spectra were acquired over the m:z range 50–550.

The total PCB concentration (tPCB) was calculated as the sum of the 23 congeners known as IUPAC# 95, 101, 136, 110, 151, 144, 149, 118, 153, 141, 138, 187, 183, 128, 174, 177, 202, 171, 180, 170, 201, 203 and 195. Identification and quantification of the individual compounds were performed by comparison with external reference standards calibrated with a 6-point calibration curve encompassing the entire concentration range. The detection limits were between 0.1 to 1 μg kg^−1^ for the PCBs standards. Analyses were performed in a series of 10 samples and 1 blank. The recoveries of the PCBs were calculated by adding known amounts of a standard to 12 homogenised replicates of the same sample; recovery levels ranged from 82–101%.

### *Marine Institute (Galway)* data

The *Marine Institute*, in Galway, Ireland examined blubber biopsies collected in 2000 from 8 BNDs from the Shannon estuary, Ireland [*see*^56^ *for Methods*].

## Data integration

A conversion factor [0.9] was generated to integrate the different sets of PCB data (*Cefas, IRBio* and *Marine Institute*) using a statistical analysis of the 13 CB congeners common to both methods [i.e. sum25CBs = sum18CBs*0.9]. The combined and converged PCB data was referred to as “sum18–25CBs (mg/kg lipid)” or \xAD\xF4PCB (Supplementary Dataset).

### Spatial and temporal trends in ∑PCB exposure

To assess the temporal trends in HP and SD data we fitted a Generalised Additive Model (GAM) to the data using the R package *mgcv*^63^. We smoothed the data using thin plate regression splines and the degree of smoothing was determined by the integrated generalised cross validation function^64^. The smoothing parameter *gamma* was set at 1.4 for HPs and set at 2.0 for SDs. To assess the spatial distribution of HP and SD data we used ∑PCB data from 1996–2012 showing spatially smoothed mean values for the ∑PCB data. This period was chosen to maximise data most representing current level of ∑PCB exposure in these species based on their temporal trends. Figures showing the spatial distribution of ∑PCBs (lipid weight) were produced in *Esri ArcMap 10.1* (www.esri.com). Data points are shown along with local averages. These averages were calculated by kernel smoothing using a polynomial order 5 kernel with power = 0, ridge parameter = 50 and bandwidth based on the spatial distribution of the observations for each species: bottlenose dolphin 0.75 degrees; harbour porpoise 0.5 degrees; killer whale 1.2 degrees; striped dolphin 0.5 degrees.

### Comparison of ∑PCBs concentrations in relation to marine mammal toxicity thresholds

Two PCB toxicity thresholds were used in this study. A lower PCB toxicity threshold was used for the onset of physiological endpoints in marine mammals of 17 mg/kg lipid weight (lw) (as *Aroclor 1254*)^7^, that was calculated to be equivalent to 9.0 mg/kg lw (as ∑PCB) in this study. A higher PCB toxicity threshold, the highest reported in marine mammal toxicology studies, of 77 mg/kg lw (as *Clophen 50*) for reproductive impairment in Baltic ringed seals (*Pusa hispida*)^6^ was calculated to be equivalent to 41 mg/kg lw (as ∑PCB) in this study. Differences for sex and cause of death in mean ∑PCBs concentrations in HPs only were investigated using univariate analysis of variance (ANOVA). Since only 4 months of ∑PCB data was available for HPs and SDs in 1990, some statistical analyses were conducted from 1991 onwards for these two species. All ∑PCB data were natural logarithm transformed (ln) prior to statistical analysis to satisfy the requirement for the residuals of ∑PCB data to be normally distributed.

We determined long-term spatial and temporal trends in ∑PCB in two cetacean data sets being UK-stranded or bycaught harbour porpoises (HPs) (n = 706) from 1990–2012 and stranded or biopsied striped dolphins (SDs) (n = 220) from 1990–2009 in the western Mediterranean Sea. Secondly, we assessed mean, sample median and population median (predicted by the geometric mean of the sample ∑PCB data) for ∑PCB exposure in male and female HPs of all ages stranded in the UK (n = 706), and stranded/biopsied SDs (n = 220), bottlenose dolphins (BNDs) (n = 131) and killer whales KWs (n = 24) from NE Atlantic and Mediterranean waters. Since our ∑PCB data is lognormal – we can use the geometric mean (GEOMEAN) as the best estimate of the population median together with upper and lower 95% CIs^10^. ∑PCB concentrations in UK HPs that were “healthy” and died of acute physical trauma (control group) (n = 345) were compared to ∑PCB concentrations in HPs that died of a range of infectious diseases (case group) (n = 252). Sex differences in ∑PCB concentrations of all species were assessed for 3 subsets of animals: sexually mature individuals only; sexually immature animals only; and those individuals where sexual maturity status was undetermined. Finally, we compared mean ∑PCB concentrations between stranded and biopsied BNDs, SDs and KWs (all HPs were stranded).

### Population Dynamics

We reviewed the current population dynamics data, mortality data (including bycatch) and reproductive status of the extant KW and other odontocete populations in European waters using strandings and necropsy data from this and other studies and systematic local and international observational surveys and other data on conservation status collected over the past 15–20 years (e.g. IUCN criteria)^8^.

### Permits

The UK Cetacean Stranding Investigation Programme conducts work on UK strandings under contract to the UK Department of Environment, Food and Rural Affairs. It has appropriate licenses from the relevant authorities (Natural England, Scottish Natural Heritage and Natural Resources Wales) to allow it to collect and hold carcasses and samples from European Protected Species, in line with UK legislation enacted under the EU Habitats Directive. The blubber samples analysed at the University of Barcelona were obtained from the biological tissue bank of the University of Barcelona (BMA Tissue Bank) and originated either from biopsies or from naturally stranded individuals. In Spain, no specific permits are required to carry out research on samples stored at public institutions. The Spanish Government translates the competency for wildlife conservation to the regional governments of the various Autonomous Communities involved in this study, and the latter provided the permits for attending strandings, obtaining tissue samples, and using them for scientific studies. All the technicians that work on marine animal strandings from the Portuguese Wildlife Society are licensed for the capture, handling, tagging and sample collection in mainland Portugal under the Decree-Law n° 140/99 of 24th April, with new redaction given by Decree-Law n° 49/2005 of 24th February and Decree-Law n° 316/89 of 22nd September. The licenses were issued by the Institute of Nature Conservation and Forestries (Government of Portugal) every year and all blubber samples were sent to the UK with the necessary CITES certification. Biopsy sampling in Slovenia was conducted under the permit of the Slovenian Environmental Agency #35601-102/2010-4. The samples in the Shannon estuary were collected under license from the National Parks and Wildlife Service.

## Additional Information

**How to cite this article**: Jepson, P. D. *et al.* PCB pollution continues to impact populations of orcas and other dolphins in European waters. *Sci. Rep.* **6**, 18573; doi: 10.1038/srep18573 (2016).

## Supplementary Material

Supplementary Dataset 1

## Figures and Tables

**Table 1:** ∑PCBs exposure (mean; sample median; geometric mean with upper and lower 95% CI; range) (mg/kg lipid) by species, region and sex (all ages).

ALL AGES	MALE							FEMALE						
Species	Mean	Median	Geomean	LowerCI	UpperCI	Min	Max	Mean	Median	Geomean	LowerCI	UpperCI	Min	Max
ALL UK Harbour porpoise (HP)	19.41	11.51	12.35	11.21	13.62	0.44	150.47	13.49	8.36	8.01	7.15	8.96	0.40	159.68
HP-Wales	22.50	16.28	14.98	12.35	18.17	0.44	150.47	12.89	8.89	8.79	7.31	10.56	1.44	86.99
HP-England	23.52	14.97	15.97	13.97	18.24	2.09	108.36	17.98	11.00	10.07	8.26	12.28	0.40	159.68
HP-Scotland	11.46	7.53	7.52	6.38	8.86	0.99	69.99	9.43	4.74	5.81	4.84	6.98	0.66	61.44
HP- Isle of Man	20.14	20.14	20.14	NA	NA	20.14	20.14	33.46	33.46	33.46	NA	NA	33.46	33.46
UK - Killer whale (KW)	107.77	107.77	67.33	8.64	524.58	23.62	191.91	241.98	110.46	126.68	42.98	373.34	33.41	819.81
KW-Ireland	NA	NA	NA	NA	NA	NA	NA	138.90	138.90	138.90	NA	NA	138.90	138.90
KW-Canary Islands	180.18	180.18	180.18	NA	NA	180.18	180.18	108.13	91.95	88.87	52.80	149.58	27.53	247.16
KW-Strait of Gibraltar	243.43	243.43	232.55	128.01	422.47	171.49	315.36	186.74	50.13	82.11	32.27	208.94	43.27	857.92
ALL UK Bottlenose dolphin (BND)	96.74	51.86	47.62	29.90	75.85	1.76	697.99	69.54	19.12	26.09	12.84	53.05	1.52	358.48
BND-England	176.94	119.54	96.60	25.43	366.95	22.13	446.55	91.17	40.61	31.26	7.93	123.24	4.11	358.48
BND-Scotland	96.61	51.86	41.59	20.23	85.49	1.76	697.99	46.13	25.40	30.14	13.31	68.21	8.53	125.08
BND-Wales	91.81	91.82	58.70	15.55	221.66	8.15	175.44	111.91	19.12	37.68	4.62	307.64	9.10	307.50
BND-Ireland	46.87	41.49	36.26	18.66	70.46	12.99	95.10	11.37	11.37	5.68	0.43	75.21	1.52	21.22
BND-Portugal	85.73	110.96	66.76	36.17	123.24	19.38	164.70	88.45	56.94	67.96	34.73	133.01	35.01	226.78
BND-Slovenia	126.83	79.58	103.79	57.53	187.26	58.00	293.23	28.64	28.64	28.64	NA	NA	28.64	28.64
BND-NW Spain	118.92	91.46	40.24	7.33	220.87	5.08	382.20	34.70	26.23	22.96	9.77	53.93	5.41	81.99
BND-Gulf of Cadiz	247.34	234.56	219.34	161.02	298.79	98.45	445.26	149.98	141.49	62.49	21.55	181.20	3.69	426.38
BND-Strait of Gibraltar	324.01	233.56	153.31	39.39	596.75	28.30	879.33	123.11	168.85	85.71	21.34	344.30	20.75	179.72
BND-Western Mediterranean	182.70	141.08	142.58	83.08	244.68	27.40	398.96	193.23	167.45	144.94	89.58	234.52	45.29	601.39
Striped dolphin (SD) -Western Mediterranean	482.15	150.43	216.91	163.78	287.28	16.80	2668.64	312.29	167.15	147.27	106.47	203.72	5.09	1518.64

**Table 2:** ∑PCB exposure (mean; sample median; geometric mean with upper and lower 95% CI range) (mg/kg lipid) by species and region (sexually matures only).

ADULTS ONLY	MALE							FEMALE						
Species	Mean	Median	Geomean	LowerCI	UpperCI	Min	Max	Mean	Median	Geomean	LowerCI	UpperCI	Min	Max
ALL UK Harbour porpoise (HP)	27.83	18.57	18.96	16.25	22.12	0.44	150.47	12.58	5.22	6.32	5.19	7.69	0.40	138.83
HP-Wales	39.85	36.46	29.19	20.49	41.57	0.44	150.47	8.97	5.12	6.07	4.51	8.17	1.44	42.16
HP-England	34.28	31.13	25.84	20.92	31.92	2.09	95.59	18.73	8.66	8.03	5.47	11.80	0.40	138.83
HP-Scotland	14.73	10.62	10.94	8.94	13.38	2.40	69.99	10.34	4.70	5.43	4.01	7.36	0.66	61.44
KW-UK	107.77	107.77	67.33	8.64	524.58	23.62	191.91	241.98	110.46	126.68	42.98	373.34	33.41	819.81
KW-Ireland	NA	NA	NA	NA	NA	NA	NA	138.90	138.90	138.90	NA	NA	138.90	138.90
KW-Canary Islands	180.18	180.18	180.18	NA	NA	180.18	180.18	108.13	91.95	88.87	52.80	149.58	27.53	247.16
KW-Strait of Gibraltar	NA	NA	NA	NA	NA	NA	NA	215.43	51.39	93.34	31.01	280.88	43.65	857.92
ALL UK Bottlenose dolphin (BND)	140.42	75.64	79.03	45.77	136.46	12.99	697.99	86.88	21.22	27.07	9.65	75.97	1.52	358.48
BND-England	446.55	446.55	446.55	NA	NA	446.55	446.55	110.12	38.95	30.35	4.11	223.88	4.11	358.48
BND-Scotland	171.88	80.31	107.36	54.85	210.16	42.44	697.99	58.63	32.34	42.11	13.84	128.17	18.46	125.08
BND-Wales	175.44	175.44	175.44	NA	NA	175.44	175.44	158.30	158.30	52.90	1.68	1665.98	9.10	307.50
BND-Ireland	46.87	41.49	36.26	18.66	70.46	12.99	95.10	11.37	11.37	5.68	0.43	75.21	1.52	21.22
BND-Portugal	103.54	110.96	86.13	34.62	214.27	34.96	164.70	61.77	61.77	56.63	24.73	129.67	37.11	86.42
BND-Slovenia	126.83	79.58	103.79	57.53	187.26	58.00	293.23	28.64	28.64	28.64	NA	NA	28.64	28.64
BND-NW Spain	236.83	236.83	186.97	46.04	759.28	91.46	382.20	5.41	5.41	5.41	NA	NA	5.41	5.41
BND-Western Mediterranean	141.08	141.08	141.08	NA	NA	141.08	141.08	NA	NA	NA	NA	NA	NA	NA
Striped dolphin (SD) -Western Mediterranean	968.55	824.91	616.89	400.47	950.26	67.59	2668.64	336.69	227.56	144.69	84.38	248.08	5.09	1518.64
